# How Political and Social Trust Can Impact Social Distancing Practices During COVID-19 in Unexpected Ways

**DOI:** 10.3389/fpsyg.2020.572966

**Published:** 2020-12-14

**Authors:** Frederike S. Woelfert, Jonas R. Kunst

**Affiliations:** ^1^ Department of Psychology, University of Oslo, Oslo, Norway; ^2^ Department of Psychology, University of Osnabrück, Osnabrück, Germany

**Keywords:** COVID-19, health policy compliance, political trust, social distancing, social trust

## Abstract

In times of the coronavirus, complying with public health policies is essential to save lives. Understanding the factors that influence compliance with social distancing measures is therefore an urgent issue. The present research investigated the role of political and social trust for social distancing using a variety of methods. In Study 1 (*N* = 301), conducted with a sample from the United Kingdom in the midst of the virus outbreak (i.e., the first wave), neither political nor social trust had main associations with self-reported social distancing tendencies. However, both factors interacted such that social trust was associated with lower social distancing tendencies among participants with low levels of political trust. In Study 2, using an experimental longitudinal design and again conducted with a sample collected from the UK (*N* = 268) during the first wave of the pandemic, social distancing practices increased over time, independent of an experimental manipulation of political trust. Moreover, while the interaction between political and social trust from the first study could not be conceptually replicated, social trust was positively related to social distancing intentions. Moving from the individual to the country level and assessing actual behavior at both the first and second wave of the pandemic, in Study 3 (*N* = 65 countries), country-level political trust was related to less social distancing during the first wave. Social trust was related to a higher growth rate of infections. Against the background of these inconsistent findings, we discuss the potential positive and unexpected negative effects of social trust for social distancing.

## Introduction

At the height of the so called “first wave” of the COVID-19 pandemic during the first quarter of 2020, almost all governments worldwide imposed lockdown policies in order to slow down the spread of the virus (Hale et al., 2020; WHO, 2020). After infection rates had slowed down over the summer, lockdown policies were loosened in many countries (Hale et al., 2020; WHO, 2020). However, since the start of September 2020, infection rates in many parts of the world rapidly increased again, initiating the “second wave” of the pandemic and further lockdowns (Hale et al., 2020; WHO, 2020). Even though most citizens seemed to abide by their government’s rules (Schreyögg et al., 2020), rallies against the lockdown policies started to grow in many countries (Carothers, 2020). The present paper aims to help understand these very different reactions to the implementation of the mentioned public health policies and compliance or non-compliance with them by focusing on the role of political and social trust.

Since the first reported case of COVID-19 in the Hubei region, China, in December 2019, the outbreak has been declared a pandemic by the World Health Organization (2020). The pandemic poses an unprecedented threat to many countries. The consequences of the outbreak have been substantial with 47.9 million COVID-19 cases worldwide and 1.2 million reported corona-related deaths, effective November 5, 2020 (WHO, 2020). Due to the lack of available treatments or vaccines, non-pharmaceutical measures to delay and mitigate the spread of COVID-19 have been implemented in most afflicted countries. Since the direct contact between humans has been identified as the most common mode of transmission, the focus on non-pharmaceutical prevention measures has mainly been to implement social distancing practices.

Social distancing (or physical distancing) refers to measures intended to increase the physical space between individuals in order to reduce the likelihood of transmissions (Gross and Padilla, 2020). These measures include but are not limited to working from home, closure of educational institutions, cancellation of mass gatherings, and “stay-at-home” policies. According to the European Centre for Disease Prevention and Control (2020b), so far, social distancing has been a key factor for reducing transmission. Nearly all governments of afflicted nations have therefore implemented policies and/or legislations to increase social distancing in order to curb the spread of the virus (Hale et al., 2020).

However, the success of these implementations relies largely on the compliance of citizens with said state measures. A large-scale pan-European survey concluded that the majority of citizens indeed support the implemented lockdown policies (Schreyögg et al., 2020). Yet, in various cities across the globe, several thousand people started to protest against the measures (Carothers, 2020). During many rallies, social distancing guidelines have been defied, causing great concern among public health experts (Gabbatt, 2020). Citizens who attend these rallies have stated a wide array of reasons for joining the protests, ranging from a concern for civil rights being restricted to believing in sundry conspiracy theories centering on the coronavirus. The reasons seem to be as manifold as the protesters themselves. Still, all converge in their common cause to oppose the implemented lockdown policies, posing the questions about potentially shared underlying factors which could impact the (non-)compliance with these measures. Simultaneously, the question of why so many other citizens are adhering to public health policies emerges. Using correlational, experimental, and longitudinal designs and focusing on processes at the individual and national level, the following studies therefore considered trust, particularly political and social trust, as potential explanatory factors for adherence to social distancing policies.

Political trust refers to citizens’ confidence in core political institutions (Zmerli, 2014). High levels of political trust have repeatedly been shown to be associated with many basic factors of well-functioning democracies such as higher political interest, and more involvement in civic affairs (Putnam et al., 1993; Zmerli, 2014). Importantly, numerous studies have indicated that higher levels of political trust are associated with higher law abidance. While most studies here focus on tax paying as a form of public policy compliance (e.g., Torgler, 2003; Alm et al., 2006; Chan et al., 2018), the investigation of the relationship between political trust and compliance with public health policies has not received as much attention. However, among the existing studies, the consensus seems to be that trust in authorities is positively related to adopting recommended or mandated preventative behavior during a pandemic (Siegrist and Zingg, 2014). Prati et al. (2011), for example, were able to show that during the H1N1 influenza pandemic, Italian citizens who trusted their health ministry were more likely to comply with the recommended health policies compared to citizens who did not trust the ministry. In line with these findings, studies conducted during the same pandemic in the Netherlands and the United States both showed that trust in the government was positively related to vaccination intentions (Quinn et al., 2009; van der Weerd et al., 2011). Blair et al. (2017) further conducted a study on the role of public trust during the Ebola epidemic in Liberia. Their results indicated that trust in the government was positively correlated with decisions to comply with mandated social distancing measures. More recently, research has shown that specific types of political trust (i.e., confidence in one’s health care system) predicted a longer lasting social distancing response, but also that general political trust may be a strong facilitator of social distancing in regions where more specific types of trust are low (Chan et al., 2020a; also see Lalot et al., 2020).

While these findings seem to suggest a consensus that trust in government positively affects compliance with preventative health measures, preliminary findings in times of the new coronavirus from a qualitative study conducted in Singapore, a country known for its high levels of political trust (Inglehart et al., 2014), indicate differently. Wong and Jensen’s (2020) analysis of their data from focus groups and social media suggested that high levels of trust in the government resulted in low compliance with the government’s health measures. The authors concluded that this may be due to the linkage of high political trust with low levels of perceived risk. In other words, if one has a high believe in one’s government solving the problem, this could theoretically also lead to passivity and a diffusion of personal responsibility. Hence, the role of political trust in the current pandemic might be less clear and it is here the present research aimed to make a contribution.

In addition to investigating the role of political trust, we also focused on the role of social trust. Health measures such as social distancing come at certain costs for the citizens, including negative impacts on their mental health due to increased social isolation (Douglas et al., 2020). At the same time, social distancing measures can only be successful if a vast majority of the population commits to their practice. We therefore argue that whether people trust other citizens and their actions might impact their engagement in social distancing practices.

Social trust, also referred to as generalized social trust, involves one’s trust in “most of the people we come across in daily life, whether we know them or not and whether they are like us or not” (Newton et al., 2018). As social trust seems to play an important role at a societal as well as at an individual level, it has received much attention from many academic disciplines (Delhey, 2014). Social trust has been found to be an important factor of social cohesion, integration, and the stability of societies (Newton et al., 2018). At the individual level, social trust has been linked to better health, happiness, prosperity, long life, and a sense of social belonging (Newton et al., 2018). Furthermore, social trust has been associated with cooperative and altruistic behavior (Uslaner, 2002; Delhey, 2014). Drawing on the literature on social capital, where social trust is often used as a key indicator, high social-trust individuals can be described as well-connected and active members of their community (Delhey and Newton, 2003; Newton et al., 2018). Trusting individuals are more likely to join voluntary associations, leading them to engage in more interactions with others compared to less trusting individuals (Stolle, 2001). By contrast, distrustful individuals tend to have less opportunity for interactions and therefore often have a smaller social network (Yamagishi, 2001).

While there seems to be a wide consensus on the positive relationship of political trust and law adherence, research on how social trust and law adherence are connected is much scarcer. As with research on political trust, studies on social trust and law adherence have mainly focused on the domain of tax compliance. However, compared to political trust, the link between social trust and tax compliance seems to be less clear-cut. Uslaner (2007) argued that since citizens are not paying their taxes to fellow citizens but rather to the state, the relationship between generalized trust and tax compliance is more complex. In his analysis of Romanian data, he found that trust is positively associated with the reported obligation of a good citizen to pay taxes (Uslaner, 2007). However, when investigating this relationship based on data of three waves from the World Values Survey, the same association could not be found. Uslaner (2007) therefore concluded that the relationship seems to be of modest size at best, and political trust plays a far bigger role in predicting tax compliance.

Regarding health-related behavior, numerous studies have been able to link social capital, and social trust specifically, to a range of positive health behaviors. For instance, high levels of social trust have been associated with non-smoking, adequate duration of sleep, and lower alcohol consumption (Lindström, 2005, 2008; Poortinga, 2006; Nieminen et al., 2013). In his review on trust and population health, Kawachi (2018) identified three mechanisms which have been proposed to link social trust to health promoting behaviors. Kawachi (2018) argued that by promoting social support, social trust can improve the access to health-relevant information, material resources and emotional support. However, it can be argued that this mechanism has the potential risk that the trusted social network can also act as a source of misinformation, which could in turn negatively affect public health (Kawachi, 2018). The author further identified a second mechanism which builds on the argument that trust can act as a facilitator of collective action (Kawachi, 2018). He argued that many measures for promoting public health (e.g., vaccinations and anti-smoking campaigns) rely on the majority of citizens to participate in said measures in order to be successful (Kawachi, 2018). Trust in fellow citizens and in their participation (as compared to free-riding) is thought to increase one’s own participation in such campaigns (Kawachi, 2018). The third mechanism Kawachi (2018) proposed, is based on the reinforcing effect that social trust is said to have on social norms. He argued that high social trust may, through a heightened adherence to social norms (e.g., washing one’s hands after using restrooms), indirectly improve public health (Kawachi, 2018).

Though the effect of social trust on general public health has previously been investigated, the effect of social trust in the context of pandemics has not received much attention. A Swedish study by Rönnerstrand (2013) intended to address this shortcoming. Rönnerstrand (2013) found that social trust was positively associated with the intent to accept vaccination against the H1N1 virus. The author proposed that this association might be due to increased altruistic tendencies in individuals with higher social trust. In line with this explanation, d’Alessandro et al. (2012) found altruistic motivations to be an important factor when deciding whether to get vaccinated against the H1N1 virus.

While these studies indicate the potentially impactful role of social trust, it is surprising how little attention it has received in the context of pandemics. The few studies that have investigated the relationship of social trust and compliance with public health policies have focused on vaccinations. While in most countries vaccinations are currently not available for the general public, it is crucial to know which factors play a role in compliance with non-pharmaceutical measures, such as social distancing. The present research therefore investigated whether social trust may play a critical part in complying with social distancing practices during the COVID-19 pandemic and consequently in mitigating the spread of the virus. As reviewed, social trust has previously been associated with positive health-related behaviors, prosocial behavior, and cooperation (Delhey, 2014). Hence, it may also be positively related to social distancing. However, since trusting individuals, compared to distrusting individuals, are more likely to engage in interactions with others (Stolle, 2001), social distancing (which limits this social tendency) could be less pronounced among them (cf. Salvador et al., 2020). Circumstantial evidence for this is also provided in a study showing that extraversion (which typically relates to more social trust; Freitag and Bauer, 2016) is related to more mobility during the COVID-19 crisis (Chan et al., 2020b).

The interrelation of social and political trust and its importance for well-functioning democracies has long been a topic of debate (Newton et al., 2018). Both forms of trust are linked to similar outcomes (e.g., low corruption, class, and education), but attempts to disentangle cause and effect of social and political trust have proven to be challenging (Newton et al., 2018). In the following, we would like to focus on the potential interaction of political and social trust in the context of compliance with public health policy.

Since people with high social trust often are well-connected and integrated within their social networks (Delhey and Newton, 2003; Newton et al., 2018), complying with social distancing measures would mean a more drastic change to these individuals’ everyday life compared to less socially trusting individuals. It is here, political trust may have a regulating function. Specifically, one could argue that socially trusting individuals would follow their tendency to frequently socialize with other people only when they at the same time show little trust in their government, including its social distancing recommendations.

In times of the coronavirus, complying with public health policies is essential to save lives. While no pharmaceutical solutions are available, social distancing seems to be one of the most promising practices to slow down the infection rate of SARS-CoV-2 and keep the healthcare systems well-functioning (European Centre for Disease Prevention and Control, 2020a,b). However, more and more people have taken to the streets to express their disagreement with the implemented lockdown policies (Carothers, 2020). If protesters were to grow in number, this could rapidly endanger the progress that has been made in terms of slowing down the spread of the virus (Gabbatt, 2020). Understanding the factors that influence compliance with social distancing measures is therefore an urgent issue.

The present research aimed to shed light on the role of political and social trust using a variety of methods. First, we examined the interplay of political trust, social trust, and social distancing at the individual-level in two samples of individuals from the UK. In the first study, we tested whether political and social trust could be associated with compliance with social distancing measures, and whether their potential effects interacted with one another. Next, in the second study, we ran a pre-registered experiment with longitudinal data in which we aimed to increase political trust and test its potential effects on social distancing. Again, we also tested for the role of social trust and its potential interaction with political trust here. Finally, moving from the individual to the national level and from self-reported to actual behavior, we tested the associations between political and social trust with behavioral social distancing at the country level at the first and second wave of the pandemic (Study 3).

## Study 1

In this first study, we tested whether political and social trust are related to self-reported compliance with social distancing measures in the UK. Furthermore, we tested for an interaction effect of social trust and political trust. The study was conducted on March 15, 2020, which was 8 days before the British government ordered their strict lockdown policies (Sparrow et al., 2020). At this time, a total of 1,391 of COVID-19 cases (Department of Health and Social Care, 2020d) and 43 COVID-19 related deaths (Department of Health and Social Care, 2020a) had been reported within the UK.

### Methods

#### Participants

Based on an a priori power simulation using the *SIMR* package (Green et al., 2016), 300 participants would provide 90% to observe a small to medium effect at a 0.05 significance level. Hence, we recruited a sample of 302 participants from the UK through the online survey platform Prolific. Participants were paid equivalent to £6.3 per hour. One participant had to be excluded due to missing data on the variables of interest, leaving a final sample of *N* = 301. The average age of the sample was 37.8 years (*SD* = 11.79) and gender was distributed nearly equally (female: 49.7%). The majority of participants reported to live in England (England: 86.4%, Scotland: 7.6%, Wales: 4.6%, and Northern Ireland: 1.0%) with 64.9% residing in an urban area and 35.1% living on the countryside. The most frequently reported ethnic/racial background was White (89.4%), followed by Black/African/Caribbean/Black British (4.0%), and Asian/Asian British (3.3%). When asked about their highest completed level of education, more than half reported having an undergraduate university degree or higher (undergraduate: 40.1%, post-graduate: 17.2%, and doctoral degree: 1.3%). A percentage of 26.8 had completed their A-levels, 13.9% their GCSEs, and 0.7% indicated primary school as their highest level of education. This and all remaining studies were conducted in compliance with the national and regional research regulations of the country of the authors’ primary affiliation.

#### Measures

##### Social Trust

Three items adopted from ESS Round 8: European Social Survey (2018) were used to measure social trust. The items are “Generally speaking, would you say that most people can be trusted or that you need to be very careful in dealing with people?,” “Do you think that most people would try to take advantage of you if they got the chance, or would they try to be fair?,” and “Would you say that most of the time people try to be helpful or that they are mostly looking out for themselves?” Items were measured on a 11-point scale ranging from 0 (*You can’t be too careful/Most people try to take advantage of me/People mostly look out for themselves, respectively*) to 10 (*Most people can be trusted/Most people try to be fair/People mostly try to be helpful*, respectively). The scale showed satisfactory internal consistency (*α* = 0.80), with higher scores indicating higher levels of social trust.

##### Political Trust

To measure participants’ political trust, the item “To which extent do you trust the government in its handling of the virus?” was used, with responses rated on a seven-point scale ranging from 0 (*Not at all*) to 6 (*Very much*).

##### Social Distancing

As in Bierwiaczonek et al. (2020), social distancing was measured by asking the participants to indicate whether they engaged in different types of social distancing behavior “as a consequence of the coronavirus outbreak”: (1) “I avoid in-person contact with others,” (2) “I avoid attending social gatherings in person,” and (3) “I try to keep a safe distance to others.” Responses were measured on a seven-point scale ranging from 1 (*strongly disagree*) to 7 (*strongly agree*). The scale showed satisfactory reliability (*α* = 0.92).

### Results

An overview of descriptive statistics and correlations for the variables social trust, political trust, and social distancing is presented in Table 1. Social trust, but not political trust, was weakly negatively correlated with self-reported social distancing.

**Table 1:** Descriptive statistics and correlations for social trust, political trust, and social distancing in Study 1.

Variable	*N*	*M*	*SD*	1	2	3
1. Social trust	301	4.94	2.02	—		
2. Political trust	301	2.04	1.58	0.19^**^	—	
3. Social distancing	301	4.36	1.63	−0.12^*^	−0.09	—

* *p* < 0.05;

** *p* < 0.01.

To test whether social and political trust function as predictors of social distancing, and more specifically, to test the hypothesis that political trust has a moderating effect on the relationship between social trust (IV) and social distancing (DV), a step-wise hierarchical multiple regression analysis was conducted. Prior to this analysis, the data was screened for outliers and influential cases. Only eight cases (3%) showed large standardized residuals (> |2|). For these cases Cook’s distances, hat values, and the covariance ratios were all within the recommended range (Field et al., 2012), resulting in the inclusion of all 301 cases in further analyses. Assumptions of linearity, randomness, normality, and homoscedasticity of residuals were examined visually, and found to be met. The Durbin-Watson test indicated that the assumption of independent errors was met.

In the first step of the regression analysis, the predictors social trust and political trust, as well as the control variables gender, age, education, income, and residence were entered. To improve interpretability (Cohen et al., 2003), the continuous variables (social trust, political trust, age, education, and income) were mean-centered, and the dichotomous variables (gender and residence) were centered by contrast coding them as -0.5 and 0.5. The regression analysis showed that for Step 1, the overall model was not significant, *F*(7, 293) = 1.84, *p* = 0.080, *R^2^* = 0.04. In the model, only the negative effect of social trust on social distancing approached significance (see Table 2). We also tested whether the influence of political and social trust may be quadratic, but both additional effects were non-significant, *ps* > 0.346. In the second step, the interaction Social Trust × Political Trust was added to the model. The interaction (see Table 2) and the overall model were significant, *F*(8, 292) = 2.56, *p* = 0.010, *R* ^2^ = 0.07, and the model led to a significant increase in explained variance compared to the first model, *F*(1, 292) = 7.33, *p* = 0.007, Δ*R^2^* = 0.02. The results for all main effects and the significant interaction effect can be found in Table 2.

**Table 2:** Hierarchical regression results on the dependent variable social distancing for Study 1.

Variable	*b*	*se_b_*	*β*	*t*	*p*
Step 1					
**Constant**	**4.32**	**0.10**		**44.38**	**<0.001**
Gender^b^	0.14	0.19	0.04	0.76	0.448
Age^a^	0.02	0.01	0.11	1.90	0.059
Education^a^	0.03	0.10	0.02	0.32	0.752
Income^a^	−0.04	0.07	−0.04	−0.63	0.528
Residence^b^	−0.30	0.20	−0.09	−1.50	0.135
Social trust^a^	−0.10	0.05	−0.12	−1.96	0.052
Political trust^a^	−0.06	0.06	−0.06	−1.05	0.296
*R^2^*	0.04				
Step 2					
**Constant**	**4.27**	**0.98**		**43.67**	**<0.001**
Gender^b^	0.11	0.19	0.03	0.60	0.548
Age^a^	0.01	0.01	0.09	1.52	0.130
Education^a^	0.02	0.10	0.01	0.18	0.856
Income^a^	−0.06	0.07	−0.05	−0.79	0.430
Residence^b^	−0.28	0.20	−0.08	−1.45	0.147
Social trust^a^	−0.07	0.05	−0.08	−1.35	0.177
Political trust^a^	−0.06	0.06	−0.05	−0.94	0.348
**Social trust** ^**a**^ × **Political trust** ^**a**^	**0.08**	**0.03**	**0.16**	**2.71**	**0.007**
***R*** ^***2***^	**0.07** ^*^				
***ΔR*** ^***2***^	**0.03** ^**^				

Statistically significant estimates are presented in bold.

* *p* < 0.05;

** *p* < 0.01.

a Mean-centered.

b Contrast coded.

**Figure 1:**
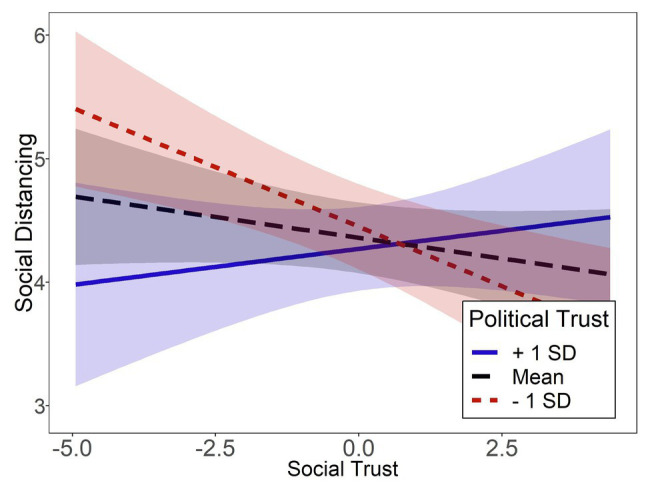
Simple slopes of social trust predicting social distancing at different levels of political trust in Study 1 are displayed. Ribbons represent 95% confidence intervals.

To follow up on the significant interaction effect, simple slopes for the predictor social trust on the dependent variable social distancing were estimated for low (1SD below mean), average (mean value), and high (1SD above mean) levels of political trust using the *interactions* R package (Long, 2019). The slopes are visualized in Figure 1. For low political trust, social trust was a significant negative predictor of social distancing, *β* = −0.24, *t*(297) = −3.20, *p* = 0.002. By contrast, at a mean level of political trust, *β* = −0.08, *t*(297) = −1.35, *p* = 0.177, and at a high level of political trust, *β* = 0.07, *t*(297) = 0.78, *p* = 0.436, the effect of social trust did not reach statistical significance.

### Preliminary Discussion

Study 1 found a weak negative correlation between social trust and self-reported social distancing. Importantly, while political trust had no main effect, it significantly moderated the effect of social trust. Specifically, whereas social trust had no association with social distancing when participants had medium or high levels of political trust, social trust negatively predicted social distancing tendencies when political trust was low. This finding might indicate that political trust indeed down-regulates the strong social tendencies of socially trusting individuals during the pandemic. While suggestive, the first study was limited primarily due to its reliance on correlational data, which prevents causal conclusions. Hence, in the next study, we aimed to replicate the results by experimentally manipulating political trust in a longitudinal test-retest design.

## Study 2

The overall goal of this study was to investigate whether an intervention aimed at increasing political trust could (a) lead to changes in social distancing, and (b) attenuate the negative effect of social trust on social distancing intentions that was observed in Study 1. In the present study, we assessed participants’ baseline political trust and social distancing. One week after, we conducted an intervention with participants who, based on the screening, had shown low levels of political trust (i.e., the group for which social trust was related to less social distancing in Study 1). Here, we first assessed their social trust and then assigned them to one of two conditions intended to alter their political trust. In the political trust condition, participants read about the high probity of politicians adopting the manipulation of a previous study (Faulkner et al., 2015). In the control condition, participants read the same text, this time framed toward bankers. Such a manipulation was chosen because politicians’ behavior can significantly influence people’s health behavior during the COVID-19 crisis (Fancourt et al., 2020). Finally, we measured participants’ social distancing intentions. Given the repeated measurement design, we were able to test whether our manipulation successfully increased political trust and social distancing from Time 1 to Time 2, and to test whether this manipulation shifted the relationship between social trust and social distancing.

This second study was conducted on March 26, 2020 (T1) and on March 30, 2020 (T2). After the British government announced a nationwide lockdown restricting people to leave home only for strictly necessary reasons such as grocery shopping, medical needs, and commuting from and to work on March 23 (Sparrow et al., 2020), a more extensive enforcement of lockdown measures by the police came into effect on March 26 (UK Home Office and Patel, 2020). By March 26 (our first measurement point), 11,658 COVID-19 cases (Department of Health and Social Care, 2020b), and 877 COVID-19 related deaths (Department of Health and Social Care, 2020a) had been registered in the UK. By March 30 (our second measurement point), 22,141 COVID-19 cases (Department of Health and Social Care, 2020c), and 2,043 COVID-19 related deaths (Department of Health and Social Care, 2020a) had been registered in the country.

### Methods

#### Participants

The present study (including power simulation, predictions, and design) was pre-registered.^1^ The sample was recruited through the online survey platform Prolific. An a priori power simulation using the *SIMR* package (Green et al., 2016) indicated that 270 participants would be needed to obtain 90% power to detect a small (*β* = 0.2) interaction involving a dichotomous predictor and a continuous moderator at a 0.05 significance criterion. As our goal was to target participants with low political trust, we originally pre-screened a total of 1,602 participants for their level of political trust. Forty-six participants had to be excluded from the sample, since they had already participated in Study 1, leaving a sample of *N* = 1,556. The sample was then divided into three approximately equally large groups based on their percentile of political trust scores. Participants who scored within the lowest third were categorized as having low political trust.

We invited these participants to partake in the second part of our study. Data from 270 participants were collected. Participants received an average reward of £9.97 per hour for participation. Two participants had to be excluded due to missing data on the variables of interest, leaving a final sample of *N* = 268 (*n*_control_ = 141, *n*_experimental_ = 127).

The average age of the sample was 36.1 years (*SD* = 12.31) and gender was distributed nearly equally (female: 49.3%). The majority of participants reported to live in England (England: 83.6%, Scotland: 11.9%, Wales: 3.0%, and Northern Ireland: 1.5%) with 81.0% residing in an urban area and 19.0% living on the countryside. The most frequently reported ethnic/racial background was White (81.7%), followed by Asian/Asian British (7.5%), multiple ethnic backgrounds (6.0%), and Black/African/Caribbean/Black British (3.0%). When asked about their highest completed level of education, more than half reported having an undergraduate university degree or higher (undergraduate: 35.4%, post-graduate: 24.3%, and doctoral degree: 2.6%). A percentage of 30.6 indicated A-levels, and 7.1% indicated the GCSEs as their highest level of education.

#### Procedure

The study consisted of two parts, the pre-screening (T1) and the experiment (T2).

##### Pre-screening (T1)

On March 26, 2020, a total of 1,602 participants were pre-screened for their level of political trust using the same item as in Study 1. Their social distancing tendency (*α* = 0.85) was also recorded so it could later be used as a baseline control in the experimental study. Only participants who scored within the lower third on the political trust item qualified for the second part and were contacted four days later for the second study. Participants were not aware of being invited to the second study based on their specific political trust scores.

##### Experiment (T2)

Participants who had been invited to participate in the second study, first, completed the measure of social trust from Study 1 (*α* = 0.86). Next, they were randomly assigned to one of two conditions. In the experimental condition, participants read a short text on the high probity of politicians, adapted from Faulkner et al. (2015). The text described the positive experiences which a fictional first-person narrator had made while working alongside politicians. In short, the narrator describes politicians with positively valanced adjectives such as genuine, honest, and sincere and that his experience is that they often are wrongfully accused of wrongdoings. In the control condition, participants read the same text with the difference that it described the high probity of bankers rather than politicians. The full texts of both conditions can be found in the Supplementary Material. In line with the procedure of Faulkner et al. (2015), in each condition, after reading one of the texts, participants were asked to name three words that were used to describe the respective group (i.e., politicians or bankers).

As a manipulation check, participants then completed the political trust item from Study 1 and then, as the dependent variable, the social distancing measure. Importantly, social distancing was measured with the same items as in Study 1, with the difference that the items were reframed to measure future intentions. Specifically, participants were asked, “As a consequence of the coronavirus outbreak, to what extent do you plan to do the following?” They then indicated their agreement with the items (1) “avoid in-person contact with others,” (2) “avoid attending social gatherings in person,” and (3) “keep a safe distance to others” on seven-point Likert scales, ranging from 1 (*Strongly disagree*) to 7 (*Strongly agree*). The scale showed satisfactory reliability (*α* = 0.88).

### Results

#### Manipulation Check

To test whether the priming of high political trust was successful, a linear mixed-effects model (LMM) was estimated. The between-subject factor condition (prime vs. control), the within-subject factor time [pre-screen (T1) vs. experiment (T2)], and the interaction of Condition × Time were all entered as fixed factors. Participants were entered as random factors. To improve the interpretability of the estimates, the variables time and condition were both centered via contrast coding (at −0.5 and 0.5) prior to analysis (Hox, 2002). The unstandardized coefficients for the main effects therefore represent the difference between the overall means of the two categories of the predictor. Assumptions of linearity and normality, and randomness of the distribution of residuals, as well as the assumption of homogeneity of variance were assessed visually and found to be met.

**Figure 2:**
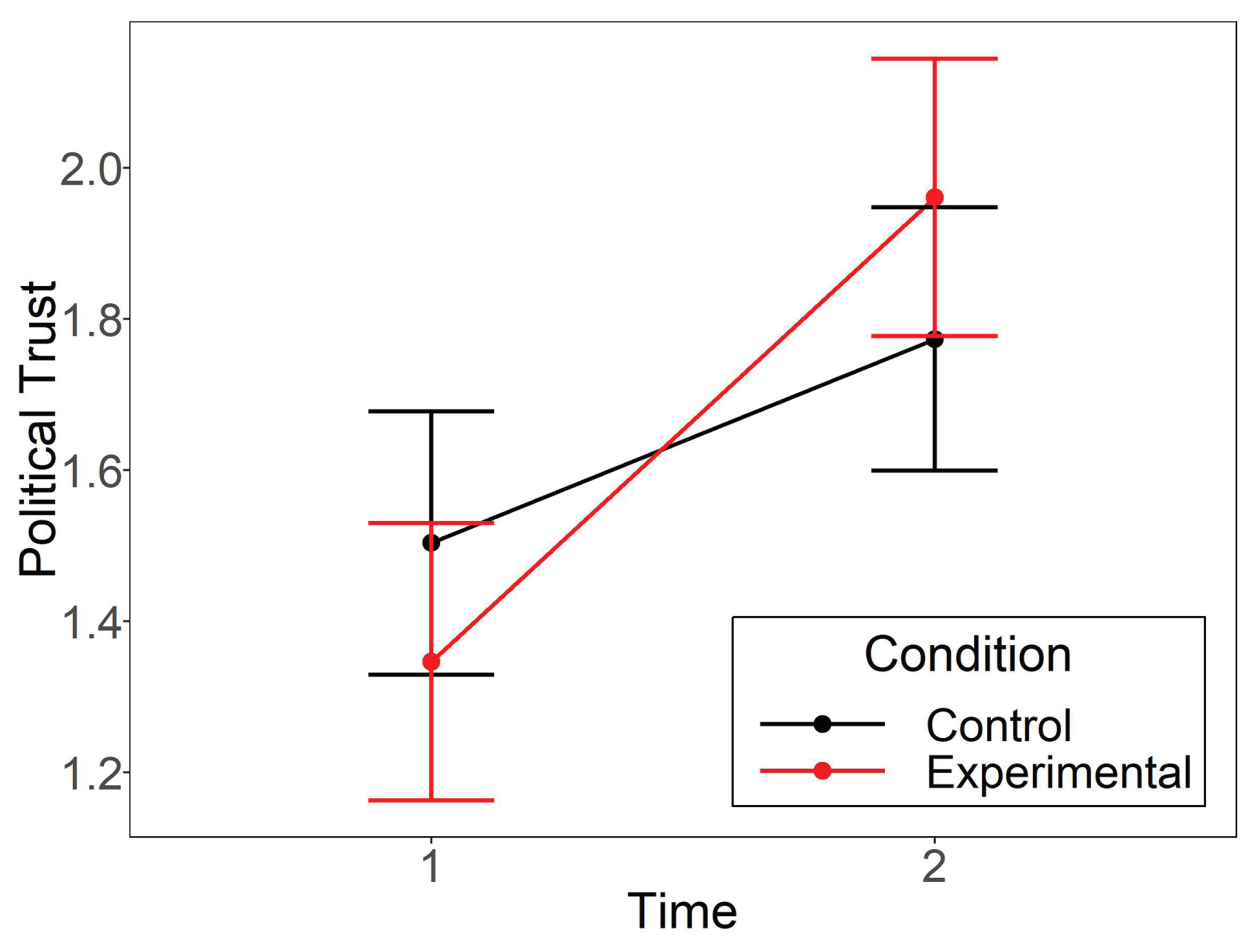
The political trust means in Study 2 at time 1 and 2 are displayed separately for the control and experimental group. Error bars represent 95% confidence intervals.

Results showed a significant effect for time, *b* = 0.44, *SE* = 0.07, *t*(266) = 6.61, *p* < 0.001, indicating that political trust increased over time, arguably due to a worsening of the situation and new state measures during the time of data collection. No main effect for condition was observed, *b* = 0.02*, SE* = 0.11, *t*(266) = 0.14, *p* = 0.890. However, the interaction between Condition × Time was significant, *b* = 0.34, *t*(266) = 2.58, *p* = 0.011. As displayed in Figure 2, there was an increase from T1 to T2 in political trust for both conditions, but this increase was more pronounced in the experimental condition, *t*(266) = −6.33, *p* < 0.001, *d* = 0.55, compared to the control condition, *t*(266) = −2.93, *p* = 0.004, *d* = 0.29. Hence, the manipulation check supported the effectiveness of the experiment in changing political trust.

#### Main Analysis

To test for main and interaction effects of the political trust manipulation and the social trust variable on social distancing, a linear mixed model was estimated in three steps (see Table 3). In line with Hox’s (2002) recommendations for improving the interpretability of the estimates, for all models, social trust (and all continuous covariates) were entered as mean-centered variables. The categorical variables condition and time, as well as the dichotomous covariates gender and residence, were centered around the theoretical mean of an equal distribution (i.e., by coding the two categories as −0.5 and 0.5). Assumptions of linearity and normality, and randomness of the distribution of residuals, as well as the assumption of homogeneity of variance were assessed visually and found to be met.

**Table 3:** Mixed linear model results predicting social distancing for Study 2.

Variable	*b*	*se_b_*	*t*	*p*
Step 1				
**Constant**	**6.69**	**0.05**	**131.73**	**<0.001**
Gender^a^ ^,^ ^c^	−0.13	0.08	−1.53	0.128
Age^a^ ^,^ ^b^	−0.00	0.00	−0.70	0.487
Education^a^ ^,^ ^b^	0.03	0.04	0.74	0.462
Income^a^ ^,^ ^b^	0.00	0.03	0.09	0.930
Residence^a^ ^,^ ^c^	0.12	0.10	1.20	0.232
**Social trust** ^**b**^	**0.04**	**0.02**	**2.17**	**0.031**
Condition^c^	−0.05	0.08	−0.56	0.573
**Time** ^**c**^	**0.13**	**0.03**	**4.09**	**<0.001**
Step 2				
**Constant**	**6.69**	**0.05**	**131.73**	**<0.001**
Gender^a^ ^,^ ^c^	−0.13	0.08	−1.59	0.114
Age^a^ ^,^ ^b^	−0.00	0.00	−0.66	0.510
Education^a^ ^,^ ^b^	0.03	0.04	0.76	0.446
Income^a^ ^,^ ^b^	0.01	0.03	0.27	0.788
Residence^a^ ^,^ ^c^	0.13	0.10	1.30	0.194
**Social trust** ^**b**^	**0.05**	**0.02**	**2.19**	**0.029**
Condition^c^	−0.05	0.08	−0.59	0.559
**Time** ^**c**^	**0.13**	**0.03**	**4.04**	**<0.001**
Social trust^b^ × Condition^c^	0.06	0.04	1.50	0.134
Condition^c^ × Time^c^	−0.05	0.06	−0.81	0.421
Social trust^b^ × Time^c^	−0.01	0.02	−0.94	0.348
Step 3				
**Constant**	**6.58**	**0.19**	**35.35**	**<0.001**
Gender^a^ ^,^ ^c^	−0.13	0.08	−1.59	0.114
Age^a^ ^,^ ^b^	−0.00	0.00	−0.66	0.510
Education^a^ ^,^ ^b^	0.03	0.04	0.76	0.446
Income^a^ ^,^ ^b^	0.01	0.03	0.27	0.788
Residence^a^ ^,^ ^c^	0.13	0.10	1.30	0.194
**Social trust** ^**b**^	**0.04**	**0.02**	**2.19**	**0.029**
Condition^d^	−0.05	0.08	−0.59	0.559
**Time** ^**c**^	**0.13**	**0.03**	**4.01**	**<0.001**
Social trust^b^ × Condition^c^	0.06	0.04	1.50	0.133
Condition^c^ × Time^c^	−0.05	0.06	−0.81	0.421
Social trust^b^ × Time^c^	−0.02	0.02	−0.95	0.342
Social trust^b^ × Condition^c^ × Time^c^	−0.01	0.03	−0.31	0.755

Statistically significant estimates are presented in bold.

a Control variable.

b Mean-centered.

c Contrast coded at −0.5 and 0.5.

As displayed in Table 3, social trust, condition, and time, as well as the control variables age, gender, education, income, and residence (urban vs. rural) were entered at Step 1. These variables were added as fixed effects, and the intercept of subjects was set to random. Results for the first step revealed that social trust and time were significant positive predictors of social distancing. There was no main effect of condition. In Step 2, the three two-way interactions Social Trust × Condition, Condition × Time, and Social Trust × Time were added. Social trust and time remained the only significant predictors in this step. In the third step, the three-way interaction Social Trust × Condition × Time was added to the model but failed to reach statistical significance.

### Preliminary Discussion

The results of Study 2 indicated that our intervention aimed at increasing political trust was successful, observing a more pronounced increase in political trust among participants of the experimental group compared to the control group. However, there was no evidence that this intervention led to changes in social distancing intentions. Instead, we found main effects for time and social trust, both of which positively predicted social distancing intentions. The main effect of time could be explained by the rapidly changing situation between the two measurement points, including the police being ordered to ensure the compliance with the lockdown polices. The positive effect of social trust was inconsistent with results from Study 1 in which social trust negatively predicted social distancing for individuals with low political trust. We argue that this different finding might be due to the differing severity of the situation and changing social norms. While social distancing was a recommended and voluntary measure when data was collected for Study 1, the situation in the UK had quickly worsened when this second study was conducted. That is, social distancing had become mandatory before the T2 data was collected and social distancing was therefore not a free choice anymore. Additionally, on the day of the first measurement point (T1), the UK government announced that the police would enforce the mandated stay-at-home policies through fines and, if necessary, arrests (UK Home Office and Patel, 2020). This policy change may have obscured potential effects of the intervention on social distancing and the influence of a potential interaction between social trust and political trust. The stricter regulations and enforcement thereof at T2 might have shifted the impact of variables of trust, toward variables of legal compliance for the decision-making process. The prevention of social isolation that might have been a motivation for individuals with low political trust and high social trust in Study 1, might be less relevant when social distancing becomes a legal issue.

One could also argue that the visibility of politicians during COVID-19 was so strong that our manipulation may lack external validity. Nevertheless, our manipulation check showed that we successfully altered political trust and research suggests that how politicians are perceived influences adherence to social distancing during COVID-19 (Fancourt et al., 2020). Yet, one could argue that such a manipulation only primes political trust for a short while and that its effect therefore may not be strong enough to alter the dependent variables.

Whereas Studies 1 and 2 provided mixed evidence that political and social trust are associated with social distancing, both studies are limited as they only focused on the individual level and self-reported social distancing. Addressing both limitations, the next study focused on the role of political trust and social trust for actual social distancing as measured through geo data on a national level. Further extending these previous studies, we included measures of the consequences of social distancing, namely, the growth rates of COVID-19 cases and COVID-19 related deaths in our analyses, in order to test whether political and social trust indirectly played a role in slowing down the spread of the virus by leading to more social distancing.

## Study 3

In the present study, we aimed at examining the main effects and interplay of political and social trust on social distancing at the country level during both main waves of the pandemic. More specifically, we investigated whether a country’s national-level political trust scores and/or social trust scores would be related to and, possibly, interactively predict its citizens’ social distancing behavior as assessed through large-scale geo data at both measured time points.

### Method

#### Sample

The sample consisted of a total of 65 countries, for which (a) political and/or social trust estimates were available through at least one of the two latest World Values Surveys (WVS; Inglehart et al., 2014; Haerpfer et al., 2020), and (b) social distancing behavioral change data through Google (2020), and/or (c) growth rates for infections and deaths from the European Centre for Disease Prevention and Control. All data sources are described in more detail below. Coronavirus data used in this study were retrieved on April 4, 2020 during the first infection wave, and on October 27, 2020 during the second infection wave.

#### Data

A complete list of the included countries, their mean level of political and social trust, their mean change of mobility, their growth rate of COVID-19 cases, and COVID-19 related deaths can be found in Supplementary Table S1 in the Supplementary Material.

##### Political and Social Trust

Data from the seventh wave of the WVS (2017–2020) was used to calculate country mean scores of political and social trust. If data from this wave was not available for a country, data from the sixth wave (2010–2014) was used. Data for both waves is publicly available at http://www.worldvaluessurvey.org. For each wave, the WVS collects representative data from a larger selection of countries. The political trust scale consisted of three items, in which participants were asked to indicate their level of confidence in (1) the government (in their nation’s capital), (2) political parties, and (3) the parliament. Answers were reported on a four-point Likert scale ranging from 1 *(A great deal)* to 4 *(None at all)*. This scale was reverse coded prior to analyses, so that higher values indicated a higher level of political trust. The scale showed high internal consistency (*α* = 0.97).

Social trust was measured combining six items from the same WVS, asking how much participants trusted people from various groups. The groups were (1) the participants’ family, (2) their neighborhood, (3) people they personally know, (4) people they meet for the first time, (5) people of another religion, and (6) people of another nationality. The four-point Likert scale ranged from 1 *(Trust completely)* to 4 *(Do not trust at all)*. This scale was reverse coded prior to analyses, so that higher values indicated higher levels of social trust. The internal consistency was satisfactory (*α* = 0.85).

##### Mobility/Social Distancing

Social distancing was assessed through the country-level COVID-19 Community Mobility Reports provided by Google (2020). Google uses GPS data to track changes in movement in different higher-level categories. These categories include Retail and Recreation, Groceries and Pharmacies, Parks, Transit Stations, Workplaces, and Residential. Changes in movement are defined as the change in the number of visits and length of stay at different places compared to a baseline. The baseline refers to the time period before the outbreak of the coronavirus (January 3–February 6, 2020). It has to be noted that the outbreak of COVID-19 was earlier in some countries, such as China. However, for most countries included in this study, the baseline period reflects the period shortly before the outbreak. All six categories were reverse scored and combined to create distancing scales with acceptable to satisfactory internal consistency for both data retrieval times (Wave 1: *α* = 0.84, Wave 2: *α* = 0.77).

##### Growth Rates of COVID-19 Cases and COVID-19 Deaths

In order to quantify how strongly countries were affected by the pandemic, the growth rates of confirmed corona cases and deaths were used. The growth rates refer to the number of days it takes for the corona cases and deaths to double in number, averaged over a 7-day period. The advantage of this measure, compared to other measures such as the total number of cases/deaths per population, is that the growth rates are not as dependent on the stage of the infection trajectory that a country is currently in. It is a more dynamic measure which allows for a cleaner interpretation of how helpful implemented restrictions, such as social distancing, have been. The growth rates for both cases and deaths were taken from the Global Change Data Lab’s project “Our World in Data” (Roser et al., 2020). Their numbers are based on daily publications of the European Centre for Disease Prevention and Control.

### Results

**Figure 3:**
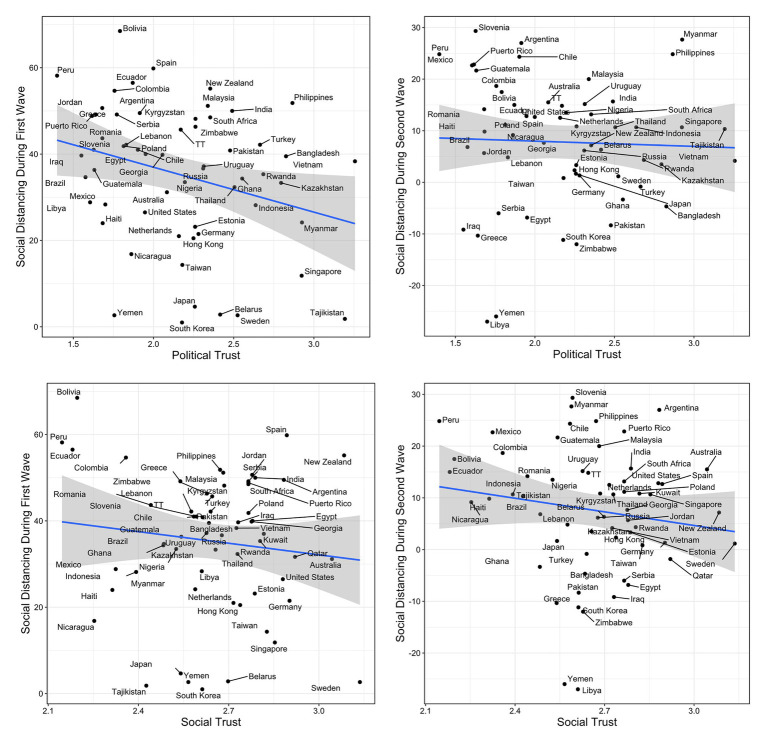
Correlation plots for political and social trust and social distancing during the first and second wave of the pandemic in Study 3. TT, Trinidad and Tobago. Ribbon represents 95% confidence intervals.

Figure 3 shows the correlation between political and social trust and social distancing during the first (April 4, 2020) and second (October 27, 2020) wave of the pandemic. Descriptive statistics and bootstrapped bivariate correlations are presented in Table 4. During the first wave, political trust was negatively correlated with social distancing, but no statistically significant relationship was observed during the second wave. In addition, social trust was positively related to the growth in infections at Wave 1. At Wave 2, this relationship turned negative and was significant by conventional *p-*value testing (*p* = 0.017) but not in terms of bootstrapped 95% confidence intervals, suggesting the influence of outliers.

**Table 4:** Descriptive statistics and correlations with 95% bootstrapped confidence intervals are presented for Wave 1 (right-hand side) and Wave 2 (left-hand side) in Study 3.

Variable	*N* ^a^	*M*	*SD*	1	2	3	4	5
1. Social trust	47	2.65	0.20	—	**0.30** **[0.06*–*0.50]**	−0.12 [−0.44 *–* 0.26]	**0.18** **[0.03*–*0.34]**	−0.01 [−18*–*0.15]
2. Political trust	45	2.17	0.44	**0.30** **[0.06 *–* 0.50]**	—	**-0.28** **[**−**0.50 *–*** −**0.04]**	0.15 [−0.14 *–* 0.42]	0.25 [−0.08 *–* 0.50]
3. Social distancing	61	35.25/7.71	15.91/12.05	−0.15 [−0.33 *–* 0.04]	−0.04 [−0.32 *–* 0.21]	—	0.04 [−0.20 *–* 0.26]	−0.30 [−0.64 *–* 0.06]
4. Growth rate – Cases	61/62^b^	8.35/160.17	4.11/172.81	−0.30 [−0.53 *–* 0.01]	0.08 [−0.15 *–* 0.37]	-0.11 [−0.34 *–* 0.08]	—	**0.46** **[0.16 *–* 0.71]**
5. Growth rate ‐ Deaths	43/54	6.35/202.90	5.36/245.36	−0.16 [−0.57 *–* 0.24]	0.03 [−0.24 *–* 0.31]	-0.06 [−0.24 *–* 0.12]	**0.60** **[0.18 *–* 0.90]**	—

a Bootstrap was conducted with 5,000 random resamples. *N*s, means and standard deviations are presented for each wave.

b Analyses with this variable excluded one extreme outlier at Wave 1 and two extreme outliers at Wave 2, see SOM. Significant estimates (*p* < 0.05) are presented in bold.

To test for main and interactive effects of political and social trust and the two waves of the pandemic on social distancing, a three-step multiple regression analysis was conducted. As for correlations, to account for the small sample size, all estimates were bootstrapped (using 5,000 re-samples). In line with recommendations to improve interpretability of the estimates (Cohen et al., 2003), the continuous predictors social trust and political trust were mean-centered whereas the variable wave was contrast coded (Wave 1 = −0.5, Wave 2 = 0.5) prior to analysis (Hox, 2002). A visual inspection of residual and scatter plots indicated that the assumptions of normality, linearity and homoscedasticity were all satisfied (Field et al., 2012). One extreme outlier (Tajikistan) was excluded from further analyses due to its large studentized residuals (−2.22), Cook’s distance (0.12) and hat (0.09) values (see Supplementary Material for details), leaving 117 observations.

In Step 1, main effects were tested by adding social trust, political trust, and wave as predictors of social distancing. In this model, wave was the only significant predictor indicating that social distancing decreased from Wave 1 to Wave 2, see Table 5. In Step 2, the two-way interactions Social Trust × Political Trust, Social Trust × Wave, and Political Trust × Wave were added to the model, but none of them reached statistical significance. Also the three-way interaction Social Trust × Political Trust × Wave, which was added in Step 3, did not reach significance. Initially, we had planned to estimate a mediation model, testing whether social distancing would mediate the association between social and political trust and growth rates. Although findings trended in the right direction (i.e., distancing being negatively related to death rates at Wave 1 and negatively to cases and death rates at Wave 2), these findings were non-significant. Hence, we decided not to test this model.

**Table 5:** Bootstrapped hierarchical regression results on the dependent variable social distancing for Study 3.

Variable	*b_original_*	*b*	*SE_b_*	*95% CI_b_*	
				*LL*	*UL*
Step 1					
**Constant**	**21.73**	**21.73**	**1.29**	**19.22**	**24.27**
Social trust^a^	−8.22	−8.00	6.64	−21.49	4.54
Political trust^a^	−3.15	−3.21	2.87	−8.73	2.54
**Wave** ^**b**^	−**28.01**	−**28.00**	**2.58**	−**33.12**	−**22.99**
Step 2					
**Constant**	**21.48**	**21.45**	**1.36**	**18.88**	**24.19**
Social trust^a^	−6.72	−6.00	7.45	−22.21	6.99
Political trust^a^	−3.09	−3.05	3.09	−9.19	2.92
**Wave** ^**b**^	−**27.87**	−**27.94**	**2.59**	−**32.94**	−**22.78**
Social trust^a^ × Political trust^a^	8.24	8.41	17.35	−25.57	42.43
Social trust^a^ × Wave^b^	−1.20	−1.41	13.93	−27.99	26.63
Political trust^a^ × Wave^b^	7.16	7.27	5.93	−4.57	18.67
Step 3					
**Constant**	**21.54**	**21.50**	**1.37**	**18.92**	**24.29**
Social trust^a^	−7.18	−6.03	7.76	−23.83	6.61
Political trust^a^	−2.93	−2.81	3.10	−9.16	3.01
**Wave** ^**b**^	−**28.56**	−**28.64**	**2.73**	−**33.90**	−**23.19**
Social trust^a^ × Political trust^a^	6.99	7.17	18.74	−30.02	43.42
Social trust^a^ × Wave^b^	2.67	2.50	15.70	−27.41	34.12
Political trust^a^ × Wave^b^	8.03	7.81	6.19	−3.94	20.31
Social trust^a^ × Political trust^a^ × Wave^b^	26.20	26.76	37.56	−47.92	99.30

*b_original_* = coefficient for linear model without bootstrapping; *b* = coefficient using bootstrapping (5,000 re-samples); *SE_b_*, bootstrapped standard error for *b*; *CI_b_*, confidence interval for *b*; *LL*, lower limit; *UL*, upper limit. Significant estimates are presented in bold.

a Mean-centered.

b Contrast coded at −0.5, 0.5.

### Preliminary Discussion

Against our expectations, the countries’ political trust scores were negatively correlated with social distancing at Wave 1. However, at Wave 2, no relationship between the variables was observed. This finding indicates that political trust, in line with preliminary qualitative evidence (Wong and Jensen, 2020), may reduce health policy compliance because it can lead to deflated risk perceptions or a false sense of security. The fact that this observation was only made at Wave 1 may be explained from a bounded rationality perspective (Simon, 1990). During the first wave, people may have experienced an overflow of novel information and, hence, heuristically relied on their governments (see Stadelmann and Torgler, 2014). At the second wave, people had several months to learn about the virus and proposed interventions, and to make up their own opinions. This process may have weakened the relationship between political trust and social distancing. Yet, given that our findings contrast with studies where political trust was a significant positive predictor of public health measures during epidemics (Quinn et al., 2009; Prati et al., 2011; van der Weerd et al., 2011; Siegrist and Zingg, 2014; Blair et al., 2017) and that the interaction between political trust and the wave of measurement was non-significant in regressions, further evidence is needed to ascertain this interpretation.

Social trust was not related to social distancing behavior. A possible reason for this could be that the measure for social distancing that was used in this study mainly captured reduction of public movement, whereas the distancing measures in the previous studies focused on contact with other people. Hence, especially the social aspect of the distancing measures may be affected by social trust. Interestingly, social trust was associated with higher infection growth rates at Wave 1. This finding once more indicates the potential negative role that social trust can play in certain health contexts. Arguably, social trust may have led to more contact with other people (something that was not directly assessed by the Google mobility data) and thereby increased the chances of infections.

## General Discussion

Social distancing policies have become a key measure in the fight against the COVID-19 pandemic around the globe. While it is important that governments introduce these measures, their success relies on the compliance of citizens. In the light of recent protests against the lockdown policies in many parts of the world, understanding the factors that influence this compliance is an urgent matter. The goal of the present research was, therefore, to examine the role of political and social trust as potential factors explaining variations in social distancing.

The first study examined the interplay of political trust, social trust, and social distancing at an individual level in the UK. Results indicated that for individuals with low political trust, social trust negatively predicted social distancing. In Study 2, we aimed to increase individual-level political trust experimentally and test its potential effects on social distancing. Whereas findings suggested that the intervention increased political trust, this did not seem to have an effect on social distancing intentions. However, social trust was positively associated with social distancing. In Study 3, we tested whether political and social trust at the country level would be related to social distancing behavior and growth rates of COVID-19 cases and deaths. Results indicated that political trust negatively predicted social distancing during the first wave of the pandemic, whereas social trust was associated with a higher growth in infection rates during the same wave.

### The Role of Political Trust

Research has shown that political trust is positively linked to compliance with health policies during epidemics (Quinn et al., 2009; Prati et al., 2011; van der Weerd et al., 2011; Siegrist and Zingg, 2014; Blair et al., 2017) including the COVID-19 pandemic (Chan et al., 2020a). We find little evidence for this in our research. Indeed, in the first two studies, political trust was not significantly related to individual-level self-reported social distancing. These findings may be explained by the fact that we measured specific trust in the government’s handling of the virus rather than general trust. Chuang et al. (2015) studied the link between different forms of social capital and health-protective behavioral intentions during a potential future influenza pandemic. The authors found that whereas general government trust positively predicted health-protective behavior, the respondents’ trust in the government’s capacity to manage the epidemic did not influence behavioral intentions (as in Studies 1 and 2). Chuang et al. (2015) argue that these two forms of trust belong to different dimensions of trust, namely, relational and calculative trust. Calculative trust describes a more rational and continual reassessment of the trustee based on the trustee’s performance (Rousseau et al., 1998; Poppo et al., 2016), whereas relational trust refers to a more stable form of trust which is mostly anchored in the past (Rousseau et al., 1998; Poppo et al., 2016). As such, relational trust, but not calculative trust may act as a form of heuristic when decisions need to be made in an uncertain situation (Rousseau et al., 1998). In support of this, Prati et al.’s (2011) study on compliance with recommendations during the H1N1 influenza pandemic showed that general trust in the ministry of health, but not the specific trust in the institutional response to the outbreak were predictive of self-reported social distancing behaviors. Together, these findings may explain why political trust in the first two studies was not related to social distancing intentions.

Against our expectations, country-level (relational) political trust was, in the third study, linked to *less* social distancing behavior during the first wave of the pandemic. The bounded rationality literature (Selten, 1990; Simon, 1990; Gigerenzer and Selten, 2002) and related research suggests that people rely on political institutions in particular when facing a higher level of information complexity (Stadelmann and Torgler, 2014). Similarly, Chuang et al. (2015) argue that in times of a pandemic, citizens get confronted with an abundance of differing information, which they often cannot fully process (Vaughan and Tinker, 2009). This reasoning may explain why political trust at the country level was associated with social distancing only during the first wave, where people did not yet have a full overview over the situation and had not fully processed the various information about it. Yet, in contrast to previous work, our results indicate that this government reliance does not have to be positive. In line with research by Wong and Jensen (2020), our study indicated that this reliance can have unintended effects, arguably because it makes citizens rely too much on their government, thereby decreasing personal attempts to reduce the spread of the virus.

### The Role of Social Trust

Considering various mechanisms, we argued that social trust could have both positive and negative associations with social distancing. On the one hand, compliance with public health policies can be interpreted as a form of altruistic practice for the common good, in which case social trust would be expected to positively relate to social distancing practices (Uslaner, 2002; Delhey, 2014). On the other hand, trusting individuals tend to engage in more social interactions compared to their more distrusting counterparts (Stolle, 2001; Yamagishi, 2001), potentially leading to a negative effect of social trust on social distancing, as the latter limits their social lives. The present studies showed some evidence for both types of relationships. In Study 1, social trust was, at the individual level, weakly and negatively related to social distancing and in Study 3 positively related to the infection growth rates at the country level during the first wave of the pandemic. Yet, in Study 2, social trust was positively related to social distancing at the individual level. We suggest that the differing individual-level results between Studies 1 and 2 may be due to the worsening of the situation in the UK during data collection, and the associated implementation of lockdown policies by the government. Arguably, this development led to a shift in social norms toward a higher compliance with social distancing. Indeed, while the mean compliance with social distancing practices was 4.36 (*SD* = 1.63) in Study 1, it was 6.65 (*SD* = 0.67) in Study 2, with the low standard deviation indicating more normativeness in Study 2 (Uz, 2014). As individuals with higher levels of social trust may be more likely to adhere to social norms (Kawachi, 2018), such a shift may have led them to show higher compliance with social distancing measures. The stricter enforcement of lockdown policies after T1 in Study 2 might also have led to a severe change of context for the decision-making process at T2. Specifically, the role of social trust when deciding whether to socially distance might have been attenuated when potential legal consequences for non-compliance were implemented.

In Study 3, social trust was positively related to infection growth rates during the pandemic’s first wave, but not to social distancing. The reason for this may be that in Study 3 social distancing was measured in terms of mobility, which does not necessarily equal contact with other people. Hence, although not observed in the present study, it is possible that social trust led to more interpersonal contact, thereby increasing infection rates. Yet, this finding would need to be investigated further with nuanced measures.

Only in the first study, did we obtain evidence for an interactive role between political and social trust for social distancing. Theoretically, social distancing in particular affects the social lives of socially trusting individuals who are well-connected to others and seek this contact. Here, political trust may serve as a behavioral regulator, such that only those who show little political trust reject social distancing. Yet, this finding could not be replicated in Studies 2 and 3, and hence needs to be interpreted with caution.

### Limitations and Future Research

The present research should be considered in the light of its limitations. Firstly, the findings from Studies 1 and 3 are based on correlational data. It is therefore not possible to ascertain inferences of causality. The longitudinal design of Study 2 aimed to address this shortcoming. Recent research suggests that the COVID-19 crisis led to more social and institutional trust (Esaiasson et al., 2020), especially among those closely affected by it (Sibley et al., 2020). This highlights the context-dependency of the effects of political and social trust on health behavior. Future research could profitably assess such changes over time by using longitudinal designs over the (ideally entire) course of a pandemic or other health crises, accounting for small and large contextual changes.

A limitation, which also should be noted, concerns the trust measures used in Study 3. For reasons of availability and accessibility, the study built on data from the WVS. We would like to stress that the WVS’ trust measures might not completely adhere with commonly used definitions of trust and trustworthiness (see e.g., Mayer et al., 1995; Rousseau et al., 1998). Future research should therefore consider the use of more refined measures, which better represent its three proposed dimensions, namely perceptions of the trustee’s (1) competence, (2) integrity, and (3) benevolence toward the trustor, as well as its two proposed higher order factors calculative/cognition-based (competence), and relational/affect-based trust (integrity and benevolence; Colquitt et al., 2007; Tomlinson et al., 2020). Moreover, one may argue that the social trust measure used may only be a distal antecedent of trust, as it mainly measures individual differences in propensity to trust, and as such may exert a limited influence especially when other trustworthiness factors are present (see Colquitt et al., 2007).

Our research was conducted in exceptional times. While governments had certain degrees of autonomy, many of their decisions were also influenced and regulated by local medical experts and international organizations such as the WHO. Thus, one can argue that the effect of political trust on social distancing may be moderated by the respondents’ perception of the medical experts and their advice. Whereas our research could only assess overall trends and patterns, future research may aim to disentangle the influence of the multitude of factors and the interactions that likely are at play.

With regards to Study 3, it should also be noted that we could have controlled for variables such as the countries’ economy, weather, and demographic structure, as was done in comparable research (Chan et al., 2020c). Yet, given that Google’s mobility data compares a country’s mobility to the country-specific baseline before the outbreak of the pandemic, the influence of these controls may be limited.

Next, Studies 1 and 2 used self-reported measures of social distancing, making responses susceptible to social desirability and other response biases. We sought to address this limitation by using geo data on actual behavior for Study 3. However, this changed the operationalization of social distancing from a more personal contact focus to a focus on public movements in general, limiting the comparability between studies. We propose that future research could address this shortcoming by assessing geo data at the individual level.

It is also important to note that the UK samples used in Study 1 and Study 2 are not be representative of the country or other afflicted areas of the world. Study 3 partially addressed this limitation by investigating the interplay of social trust, political trust, and social distancing across countries with data derived from representative samples. Yet, future studies conducted within and across countries with representative samples are needed.

## Conclusion

The present research examined the interplay of political trust, social trust, and social distancing during the COVID-19 pandemic at different levels of analysis and using a variety of methods. Findings indicated that both political and social trust can have unexpected effects on compliance with social distancing policies. Political trust may lead to an overreliance on the government, thereby decreasing personal efforts to combat the pandemic. Social trust may in an altruistic manner lead to more social distancing, but may also impair adherence to such measures, possibly especially when trust in the government’s handling of the situation is low.

## Data Availability Statement

The datasets presented in this study can be found in online repositories. The names of the repository/repositories and accession number(s) can be found at: https://osf.io/78gr4/files/?view_only=49f44ea1df9f46f5bae3faef93575488, https://www.google.com/covid19/mobility/, and http://www.worldvaluessurvey.org.

## Ethics Statement

Ethical review and approval was not required for the study on human participants in accordance with the local legislation and institutional requirements. The participants provided their written informed consent to participate in this study.

## Author Contributions

FSW and JRK designed the studies and collected the data. FSW conducted the analysis and drafted the first version of the manuscript. JRK helped with the analyses and provided critical revisions of the draft. All authors contributed to the article and approved the submitted version.

### Conflict of Interest

The authors declare that the research was conducted in the absence of any commercial or financial relationships that could be construed as a potential conflict of interest.

## Footnotes

^1^see https://osf.io/nrqfw/?view_only=0aaaa6e351074f52be1de6289f2657c3
