# Supplementary material for: How Political and Social Trust Can Impact Social Distancing Practices During COVID-19 in Unexpected Ways
Source: Front Psychol. 2020 Dec 14;11:572966. doi: 10.3389/fpsyg.2020.572966 (PMC7767922; doi:10.3389/fpsyg.2020.572966)
Supplement: Supplementary file 1 [file Data_Sheet_1.pdf]

Supplementary Material

Texts Used for Priming High and Neutral Political Trust

Prime for High Political Trust

A poll released by the market research firm IPSOS in 2018 found that politicians were among the least trusted professionals in Britain. Only few of the respondents said that they would trust government ministers and politicians in general to tell the truth.

It has become fashionable to call politicians distrustful. However, my experience of working alongside senior politicians from both major parties left me with a very different view. It is worth pointing out that politicians work significantly longer hours and get paid considerably less than their friends in the private sector. And they face intense and, increasingly, vindictive media scrutiny. Numerous fact-checking organisations have shown that while the media report the negative cases, the vast majority of statements politicians make are accurate and truthful.

It is also worth remembering that when people say they hate politics, they do not really mean it. They mean rather that they hate forms of politics. Public servants and politicians are working quietly and typically without thanks doing positive things for the public behind the scenes every day. In other words, we should not forget that, beyond the sound and fury of partisan politics, a lot of public policy goes on in the background. This ranges from the effective delivery of many healthcare services, education, and delivering critical infrastructure like the maintenance of roads and regulation of water. We often take these things for granted but for this we really should be thankful.

While a very small minority of politicians may be engaged in corrupt acts, the vast majority are working tirelessly to achieve successful public policy outcomes that often go unrecognised precisely because they are so successful. In fact, the likelihood of a randomly selected politician being engaged in corruption is almost zero.

Although it is not reflected in public discussion and media coverage, using words like truthful, honest, sincere, loyal and genuine to describe most politicians and public servants is not as ridiculous as it may sound. I may be criticised for saying so, but I can honestly say these words best describe my experience working alongside politicians from both major parties.

Neutral Prime

A poll released by the market research firm IPSOS in 2018 found that bankers were among the least trusted professionals in Britain. Only few of the respondents said that they would trust bank managers and bankers in general to tell the truth.

It has become fashionable to call bankers distrustful. However, my experience of working alongside senior bankers from different major banks left me with a very different view. It is worth pointing out that bankers work significantly longer hours and get paid considerably less than professionals in other sectors. And they face intense and, increasingly, vindictive media scrutiny. Numerous fact-checking organisations have shown that while the media report the negative cases, the vast majority of statements bankers make are accurate and truthful.

It is also worth remembering that when people say they hate the banking sector, they do not really mean it. They mean rather that they hate certain forms of banking. Bankers are working quietly and typically without thanks doing positive things for the public behind the scenes every day. In other words, we should not forget that, beyond the negative image of the banking sector, a lot of policy goes on in the background. Collectively banks carry out a wide range of roles from personal finance to raising funds for governments and underwriting issues of bonds and shares. They are essential for businesses and the economy. We often take these things for granted but for this we really should be thankful.

While a very small minority of bankers may be engaged in corrupt acts, the vast majority are working tirelessly to achieve successful outcomes for stakeholders of the private and public sector that often go unrecognised precisely because they are so successful. In fact, the likelihood of a randomly selected banker being engaged in corruption is almost zero.

Although it is not reflected in public discussion and media coverage, using words like truthful, honest, sincere, loyal and genuine to describe most bankers is not as ridiculous as it may sound. I may be criticised for saying so, but I can honestly say these words best describe my experience working alongside bankers from all major banks.

Descriptive Statistics for Countries Included in Study 3

Table S1

Countries and their Mean Levels on Variables of Interest in Study 3

Country	Political Trust	Social Trust	Distancing		Growth Rate Cases		Growth Rate Deaths	
			Wave 1	Wave 2	Wave 1	Wave 2	Wave 1	Wave 2
Argentina	1.91	2.88	49.50	27.00	8.00	51.21	6.24	52.53
Australia	2.08	3.04	31.17	15.50	9.78	104.10	5.80	-
Bangladesh	2.83	2.63	39.50	-4.67	20.24	190.90	26.61	203.62
Belarus	2.42	2.70	2.83	6.33	3.68	81.48	-	164.09
Bolivia	1.79	2.20	68.50	17.50	7.70	641.55	-	266.85
Brazil	1.58	2.48	34.67	6.83	4.98	162.52	3.56	234.66
Chile	1.90	2.58	41.00	24.33	5.76	234.96	3.27	205.34
Colombia	1.76	2.36	54.67	18.67	5.68	81.61	3.40	115.73
Cyprus	2.10	2.62	-	-	5.43	16.04	8.25	-
Ecuador	1.87	2.18	56.50	15.00	6.67	87.43	3.84	340.29
Egypt	1.95	2.78	40.00	-6.83	10.70	443.90	6.28	-
Estonia	2.26	2.79	23.17	3.33	9.45	60.18	1.95	68.38
Ethiopia	2.44	2.73	-	-	6.20	115.75	-	94.39
Georgia	2.06	2.78	39.83	7.67	8.08	8.93	-	9.52
Germany	2.28	2.90	21.50	1.33	8.53	26.14	3.82	184.57
Ghana	2.55	2.48	34.33	-3.33	12.04	572.77	9.50	253.11
Greece	1.64	2.54	49.17	-10.33	9.46	24.33	6.51	43.74
Guatemala	1.63	2.54	36.33	21.67	10.87	152.02	-	158.60
Haiti	1.68	2.31	24.00	9.83	5.98	873.46	-	1123.24
Hong Kong	2.25	2.74	20.50	2.33	-	-	-	-
India	2.49	2.79	50.00	15.67	4.04	107.92	3.81	132.25
Indonesia	2.64	2.39	28.17	10.67	7.57	66.39	6.62	79.50
Iran	2.45	2.72	-	-	10.90	66.89	17.07	68.89
Iraq	1.55	2.73	39.67	-9.17	9.29	86.94	16.17	143.82
Japan	2.26	2.54	4.67	1.67	7.22	115.29	14.18	168.37

Country	Political Trust	Social Trust	Distancing		Growth Rate Cases		Growth Rate Deaths	
			Wave 1	Wave 2	Wave 1	Wave 2	Wave 1	Wave 2
Jordan	1.68	2.78	50.67	5.67	14.11	14.01	-	9.78
Kazakhstan	2.80	2.66	33.33	3.50	5.59	353.55	-	292.87
Kuwait	-	2.82	37.00	10.83	11.59	105.76	-	90.74
Kyrgyzstan	2.26	2.69	48.17	10.83	5.34	69.46	-	237.21
Lebanon	1.83	2.58	42.17	4.83	20.75	35.42	5.87	50.54
Libya	1.70	2.61	28.33	-27.00	2.02	35.69	-	53.86
Malaysia	2.34	2.68	51.17	20.00	11.20	18.41	6.81	22.38
Mexico	1.60	2.32	28.83	22.67	6.66	105.08	3.01	150.28
Myanmar	2.93	2.59	24.17	27.67	3.50	22.41	-	23.66
Netherlands	2.16	2.72	21.00	12.50	8.05	19.86	4.84	110.64
New Zealand	2.35	3.08	55.17	7.17	7.10	139.98	-	-
Nicaragua	1.86	2.25	16.83	9.17	5.30	323.07	-	749.64
Nigeria	2.20	2.53	33.50	13.50	5.69	542.53	7.00	782.21
Pakistan	2.48	2.61	40.83	-8.33	7.47	267.39	3.92	378.11
Panama	1.40	2.15	58.17	24.83	5.27	216.93	2.83	437.69
Philippines	2.87	2.67	51.83	24.83	3.66	142.26	5.25	91.38
Poland	1.81	2.77	41.83	11.17	5.45	13.03	3.26	22.52
Puerto Rico	1.62	2.77	48.83	22.83	2.73	56.76	2.41	105.92
Qatar	-	2.92	31.67	-1.83	9.26	359.70	-	183.56
Romania	1.68	2.44	43.67	14.17	5.38	32.30	2.83	55.78
Russia	2.31	2.68	36.67	6.17	3.50	61.64	2.27	64.52
Rwanda	2.69	2.80	35.33	4.33	9.71	301.45	-	-
Serbia	1.77	2.77	49.17	-6.00	4.14	52.05	2.59	254.08
Singapore	2.92	2.85	11.83	10.67	11.55	4847.35	5.30	-
Slovenia	1.63	2.59	41.00	29.33	12.42	8.58	6.08	35.53
South Africa	2.35	2.77	48.50	13.17	19.27	299.85	3.87	176.30
South Korea	2.18	2.61	1.00	-11.17	70.23	175.54	23.52	169.25
Spain	2.00	2.89	59.83	12.67	10.49	39.27	5.98	154.98

Country	Political Trust	Social Trust	Distancing		Growth Rate Cases		Growth Rate Deaths	
			Wave 1	Wave 2	Wave 1	Wave 2	Wave 1	Wave 2
Sweden	2.52	3.14	2.67	1.17	7.41	56.98	4.28	1197.64
Taiwan	2.18	2.83	14.33	.83	17.03	264.43	5.30	-
Tajikistan	3.19	2.43	1.83	10.33	-	181.11	-	390.58
Thailand	2.51	2.73	32.33	10.67	8.75	392.69	3.63	-
Trinidad and Tobago	2.17	2.65	45.67	14.83	11.68	110.87	4.42	54.68
Tunisia	1.51	2.56	-	-	6.98	23.78	4.42	13.54
Turkey	2.66	2.64	42.17	-.83	3.73	119.53	3.17	92.80
United States	1.95	2.88	26.50	12.83	4.97	83.78	3.39	193.08
Uruguay	2.31	2.63	37.17	15.17	10.93	42.19	-	84.89
Vietnam	3.26	2.73	38.33	4.17	14.31	193.15	-	-
Yemen	1.76	2.57	2.67	-26.00	-	3334.15	-	-
Zimbabwe	2.26	2.63	46.33	-12.00	8.25	277.33	-	114.98

Note. Political and social trust scores were calculated using data from the World Values Survey 6 and 7 (2010-2014; 2017-2020). Distancing scores were calculated using the mobility change data as provided by Google (2020). The growth rates refer to the number of days it took for the COVID-19 cases and deaths to double in number, averaged over a seven-day period. Data for Wave 1 was retrieved April 4, 2020. Data for Wave 2 was retrieved October 27, 2020.

Exclusion of Cases for Correlation Analyses in Study 3

Figure S1

Scatterplots Showing Extreme Outliers for Relationships with Infection Rates

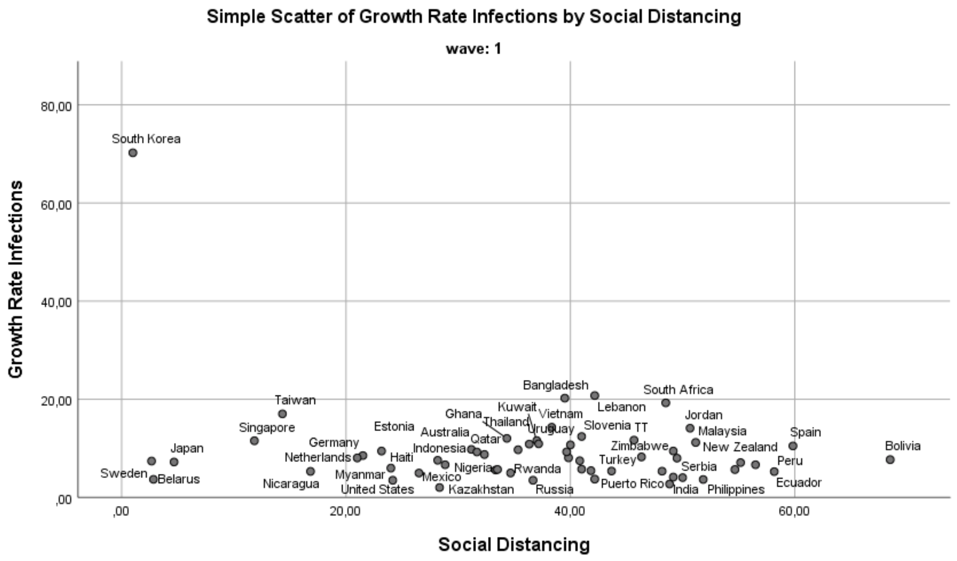

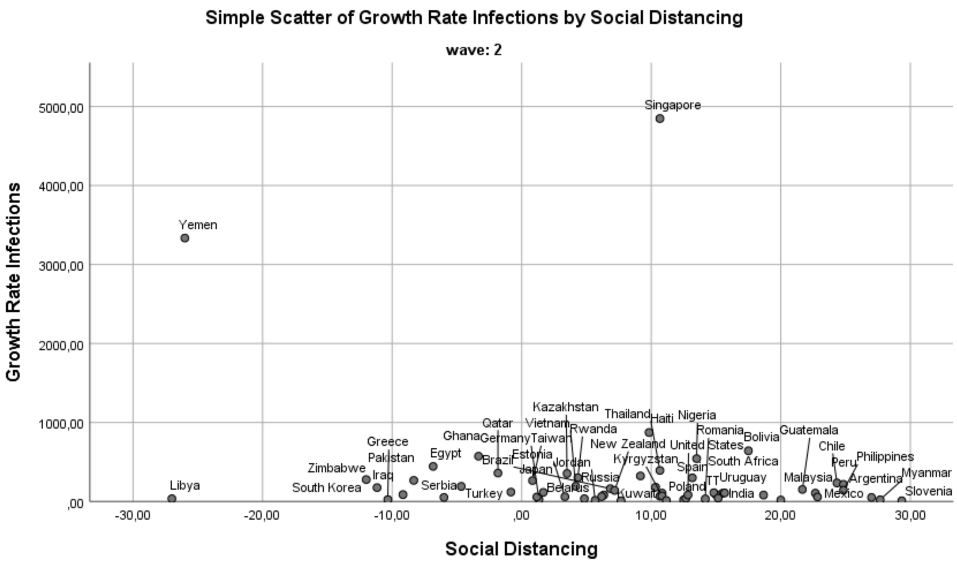

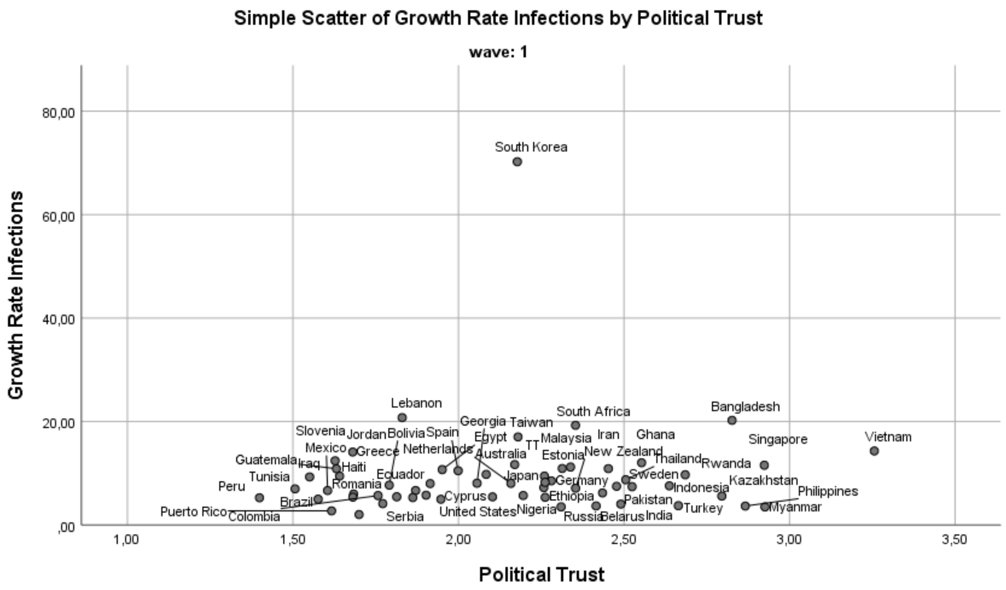
Simple Scatter of Growth Rate Infections by Political Trust
wave: 1
Growth Rate Infections
Political Trust
80,00
60,00
40,00
20,00
,00
1,00
1,50
2,00
2,50
3,00
3,50
South Korea
Lebanon
Georgia
Taiwan
South Africa
Bangladesh
Slovenia
Jordan
Bolivia
Spain
Egypt
Malaysia
Iran
Ghana
Singapore
Vietnam
Mexico
Greece
Netherlands
Australia
TT
Estonia
New Zealand
Guatemala
Iraq
Haiti
Thailand
Rwanda
Tunisia
Ecuador
Japan
Germany
Sweden
Kazakhstan
Peru
Romania
Indonesia
Philippines
Cyprus
Ethiopia
Pakistan
Brazil
Puerto Rico
Colombia
Serbia
United States
Nigeria
Russia
Belarus
India
Turkey
Myanmar

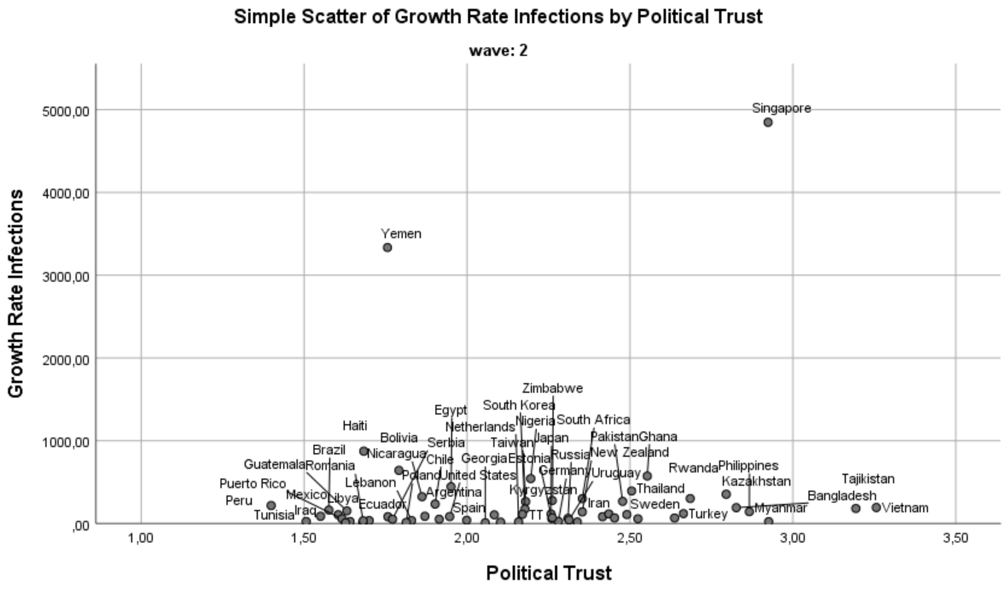
Simple Scatter of Growth Rate Infections by Political Trust
wave: 2
Growth Rate Infections
Political Trust
5000,00
4000,00
3000,00
2000,00
1000,00
,00
1,00
1,50
2,00
2,50
3,00
3,50
Singapore
Yemen
Zimbabwe
Egypt
South Korea
Haiti
Netherlands
Nigeria
South Africa
Bolivia
Japan
Pakistan
Ghana
Brazil
Nicaragua
Serbia
Taiwan
Russia
New Zealand
Guatemala
Romania
Chile
Georgia
Estonia
Rwanda
Philippines
Puerto Rico
Lebanon
Poland
United States
Germany
Uruguay
Kazakhstan
Tajikistan
Mexico
Libya
Argentina
Kyrgyzstan
Thailand
Bangladesh
Peru
Ecuador
Spain
Iran
Sweden
Myanmar
Vietnam
Iraq
Tunisia
TT
Turkey

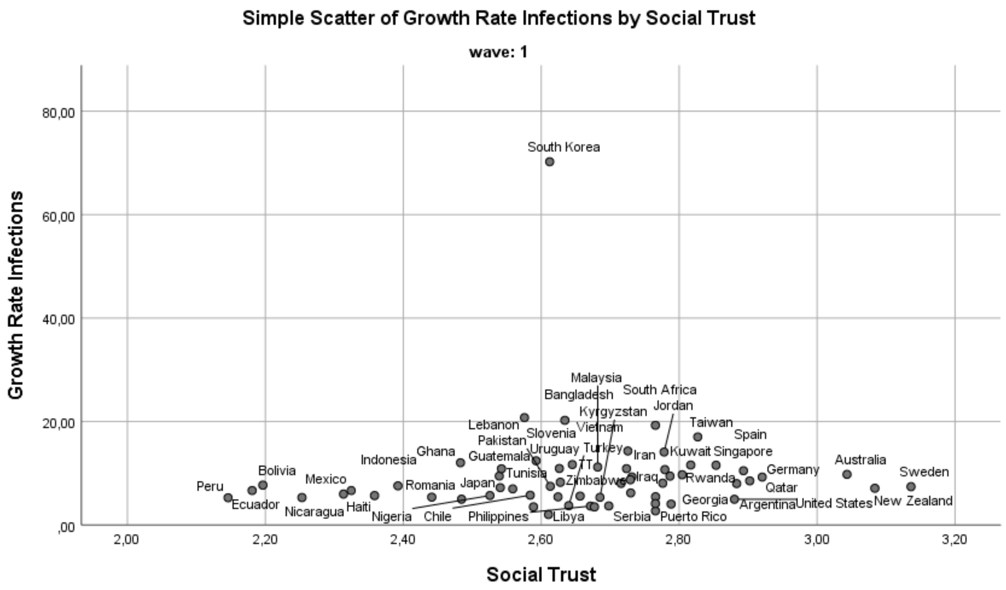
Simple Scatter of Growth Rate Infections by Social Trust
wave: 1
Growth Rate Infections
80,00
60,00
40,00
20,00
,00
2,00
2,20
2,40
2,60
2,80
3,00
3,20
Social Trust
South Korea
Malaysia
Bangladesh
South Africa
Kyrgyzstan
Jordan
Lebanon
Vietnam
Taiwan
Slovenia
Spain
Pakistan
Uruguay
Turkey
Iran
Kuwait
Singapore
Ghana
Guatemala
Australia
Indonesia
TT
Germany
Sweden
Bolivia
Tunisia
Iraq
Mexico
Romania
Japan
Zimbabwe
Rwanda
Qatar
Peru
Georgia
Ecuador
Haiti
Argentina
United States
New Zealand
Nicaragua
Nigeria
Chile
Philippines
Libya
Serbia
Puerto Rico

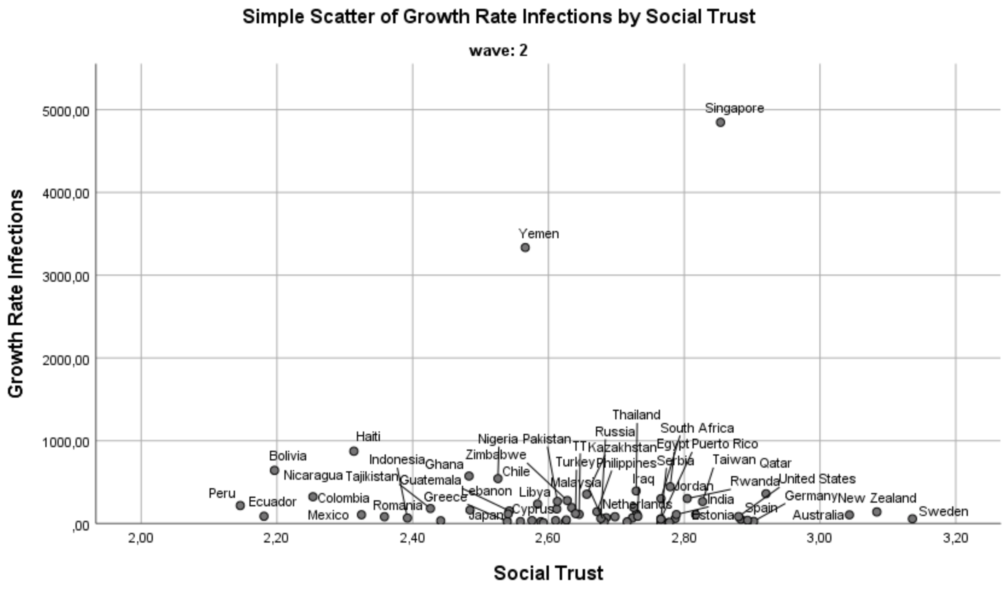
Simple Scatter of Growth Rate Infections by Social Trust
wave: 2
Growth Rate Infections
5000,00
4000,00
3000,00
2000,00
1000,00
,00
2,00
2,20
2,40
2,60
2,80
3,00
3,20
Social Trust
Singapore
Yemen
Thailand
South Africa
Russia
Haiti
Nigeria
Pakistan
TT
Kazakhstan
Egypt
Puerto Rico
Bolivia
Zimbabwe
Indonesia
Ghana
Turkey
Philippines
Serbia
Taiwan
Qatar
Chile
Nicaragua
Tajikistan
Guatemala
Malaysia
Iraq
Rwanda
United States
Peru
Lebanon
Libya
Jordan
Colombia
Greece
Germany
New Zealand
Ecuador
Romania
Netherlands
India
Spain
Mexico
Japan
Cyprus
Estonia
Australia
Sweden

Exclusion of Cases for Regression Analyses in Study 3

Figure 1

Studentized Residuals vs. Leverage Plot for Regression Analyses in Study 3

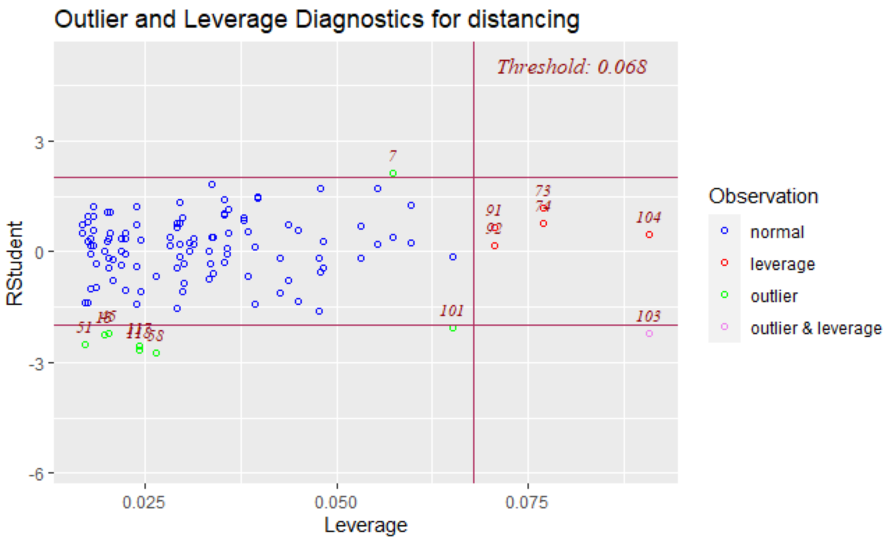

Note. The graph shows the studentized residuals (y-axis) and the leverage, as represented by hat values (x-axis) for the linear regression model of social distancing (DV) with political trust, social trust, and wave as predictors. Case 103 represents the data of Tajikistan during the first wave which was excluded from further analyses.

Figure 2

Bar Plot of Cook's Distance for Regression Analyses in Study 3

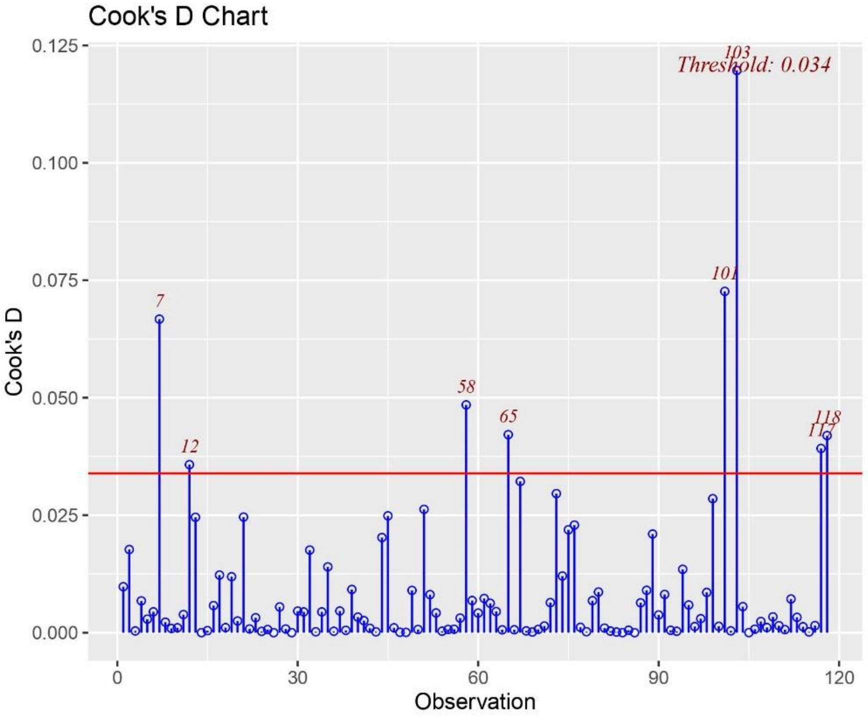

Note. The bar plot shows the Cook’s distance values for the country sample of Study 3 for the linear regression model of social distancing (DV) with political trust, social trust, and wave as predictors. Case 103 represents the data of Tajikistan during the first wave which was excluded from further analyses.
